# 中国天津大港油田LDCT肺癌筛查人群防治知识调查与分析

**DOI:** 10.3779/j.issn.1009-3419.2014.02.16

**Published:** 2014-02-20

**Authors:** 冠华 任, 剑飞 叶, 亚光 范, 竞 王, 志娟 孙, 辉 贾, 欣欣 杜, 朝华 候, 颖 王, 永成 赵, 清华 周

**Affiliations:** 1 300192 天津，天津市核医学重点实验室，中国医学科学院放射医学研究所，北京协和医学院 Peking Union Medical College & Institute of radiation medicine, Chinese Academy of Medical Science, Tianjin 300192, China; 2 300280 天津，大港油田总医院 Department of Thoracic Surgery, Dagang Oil Field General Hospital, Tianjin 300280, China; 3 300052 天津，天津市肺癌转移与肿瘤微环境重点实验室，天津肺癌研究所，天津医科大学总医院 Tianjin Key Laboratory of Lung cancer Metastasis and Tumor Microenvironment, Tianjin Lung cancer Institute, Tianjin Medical University General Hospital, Tianjin 300052, China; 4 天津医科大学总医院放射科 Department of Radiology, Tianjin Medical University General Hospital, Tianjin 300052, China

**Keywords:** 肺癌防治, 知晓率, 影响因素, Lung Cancer prevention and control, Awareness, Affecting factors

## Abstract

**背景与目的:**

已有的研究表明提高人群对肺癌防治知识的认识水平，有助于肺癌高危人群肺癌筛查项目的参与度。本研究的目的是调查卫生部肺癌早诊早治大港项目点低剂量螺旋（low dose CT, LDCT）肺癌筛查人群肺癌防治知识知晓情况及个体因素对其的影响，为肺癌高发现场的综合防治提供依据。

**方法:**

通过整群抽样和自愿参加方法对参加LDCT筛查的大港油田肺癌高发现场职工进行问卷调查。

**结果:**

本次调查共获得有效问卷1, 633份，调查对象的平均年龄为60.08±6.58，男性1, 343人（82.2%），女性290（17.8%）。对肺癌的知晓率、危险因素、筛查方法，体检意愿以及治疗的知晓率分别为：64.5%、77.1%、43.7%、49.6%、52.8%。多因素*Logistic*回归分析结果表明：教育、年龄、吸烟包年、疾病史是调查对象肺癌防治知识知晓的影响因素，教育和年龄的OR值分别为0.567（95%CI: 0.439-0.733）和1.373（95%CI: 1.084-1.739）。调查人群中80.3%的人群能接受1年1次的体检，人群体检费用承受能力不高。对被调查者体检意愿进行多因素分析得出，性别、年龄、癌症知识知晓情况以及家庭年平均收入是筛查意愿的影响因素。

**结论:**

教育程度和吸烟影响人群对肺癌防治知识的认知情况，应加强对低教育水平人群的癌症健康教育。在肺癌高发现场，肺癌的筛查应与戒烟和健康教育紧密结合，实行肺癌的综合防治。

## Abstract (en)

**Background and objective:**

It has been proven that increase of the awareness level of lung cancer prevention and control could enhance participation of lung cancer screening of lung cancer high risk group. The aim of this study is to investigate the awareness level of lung cancer prevention and control and the effect of individual characteristics on lung cancer awareness, and to provide evidence for comprehensive lung cancer prevention in high risk areas of lung cancer.

**Methods:**

Staffs of Tianjin Dagang Oil Field who participate low dose CT (LDCT) lung cancer screening by cluster sampling or according to voluntary principle were surveyed, data of lung cancer awareness were collected by questionnaire.

**Results:**

A total of 1, 633 valid questionnaires were collected. The average age of respondents was 60.08±6.58. Most participants were males (82.2%) while female only accounted for 17.8%. The proportions of awareness about lung cancer in China, risk factors, screening methods and the knowledge of health examination were 64.5%, 77.1%, 43.7%, 49.6% respectively. Result of multiple logistic regression analysis showed that education level, smoking (pack-year), age, prior tuberculosis were the influencing factors of lung cancer awareness with adjusted Ors for education and age level as of 0.567 (95%CI: 0.439-0.733) and 1.373 (95%CI: 1.084-1.739) respectively. 80.3% of the participants can accept health examination once a year, while the ability to pay the medical expenses was not high. The influencing factors of health examination willingness were gender, age, income, the knowledge of lung cancer.

**Conclusion:**

Education level and smoking affect the awareness of lung cancer prevention and control, health education for lung cancer should be conducted especially in population with low education level. Comprehensive lung cancer control in high risk areas should combined lung cancer screening, tobacco control and health education.

据世界卫生组织估计，2008年肺癌占全球1, 270万例新发癌症和760万癌症死亡的13%和18%，是男性死亡和发病的首要因素^[1]^。2012年美国新增肺癌病例22万，死亡16万^[2]^。2009年陈万青等报道全国肿瘤登记地区恶性肿瘤发病率，肺癌居于首位，结果表明在中国，无论是男性还是女性，城市或乡村，肺癌死亡率也居癌症死亡的首位^[3, 4]^。肺癌是目前中国人群面临的比较严峻的癌症类型，早期肺癌并没有明显的临床症状，而大部分患者在有临床症状时才去就诊，但病变确诊后，75%-80%为中晚期肺癌，而其中仅有不到25%的患者适合手术治疗^[5]^。自2010年起，肺癌筛查被纳入卫生部癌症早诊早治项目，在天津大港、云南宣威的高危人群开展了利用低剂量螺旋CT（low dose CT, LDCT）进行肺癌的年度性筛查^[6, 7]^。本研究对2010年- 2011年大港项目点基线信息中的人群肺癌防治知识进行了分析，以期了解筛查人员对肺癌知识的知晓率以及影响因素，从而促进肺癌健康教育和早期诊断的有机结合。

## 1 对象与方法

### 1.1 调查对象

研究对象为2010年1月-2011年6月期间肺癌早诊早治项目天津大港项目点参加LDCT筛查的大港油田职工。利用整群抽样和自愿原则选取50岁-74岁且有20包年及以上吸烟史的大港油田职工作为筛查对象。共录入问卷1, 807份，其中有效问卷1, 633份，有效率90.4%。

### 1.2 调查内容与方法

问卷由卫生部癌症早诊早治项目专家组制定（此问卷于2011年进行了修订，此处使用修订前版本）。基线调查中的肺癌防治知识问卷包括肺癌的基本认识、肺癌危险因素、肺癌筛查方法、肺癌治疗等内容。调查员均经培训和考核合格后参与调查。数据录入采用Visual Foxpro软件双人独立录入，利用epidata进行一致性检验，然后再利用Visual Foxpro进行逻辑性检验。

### 1.3 统计分析

选用描述性统计方法计算人群的肺癌防治知识知晓情况，利用*χ*^2^检验比较不同人群特征的知晓率之间的差别。对肺癌防治知识进行量化，0为知晓9个或更少选项，1为知晓9个以上的选项进行单因素与多因素*logistic*分析，检验水准为*α*=0.05，知晓率=调查知晓人数/总调查人数。对体检意愿使用同样方法进行量化，将接受2年以上体检的人群设为1，接受1年1次和2年1次体检的人群设为0，进行多因素分析。

## 2 结果

### 2.1 调查对象的一般情况

本次问卷调查问卷为1, 807份，其中有效问卷1, 633份，有效率81.65%。本次调查对象的平均年龄为60.08±6.58，55岁及以下占28.7%，55岁以上占71.3%，调查对象中男性1, 343人（82.2%），女性290（17.8%）。71.4%的调查对象吸烟包年（每日的吸烟包数乘以吸烟的年数）在30以上，吸烟包年在29以下所占比例为28.6%。文化程度根据受教育水平分为5个等级，以初中水平所占比例最大（41.2%），其次为高中、小学、中专以上，未正式上过学分别为23.5%、19.1%、13.3%、2.8%。76.3%的人群饮食为荤素平均，单纯食素和食肉分别占10.5%和13.2%。此次调查中80%以上的人群有饮酒史，偶尔饮酒和经常饮酒分别占44.7%和33.5%, 重度饮酒者不到1%。19.5%的个体有癌症家族史，有肺部疾病史的个体不足10%，有心脏疾病史、胃肠疾病史、糖尿病史的比例分别是29.9%、16.9%、15.9%。

### 2.2 肺癌相关防治知识总体知晓率情况

肺癌相关防治知识调查包括：对肺癌的基本认识、肺癌危险因素，早期肺癌检测方法、肺癌治疗的认识情况，以及筛查意愿和拒绝筛查的原因。大港油田的工人对肺癌的一般认识较差，总体知晓率仅有64.5%，其中肺癌的早期阶段知晓率较高为93%，对普查以及肺癌预后生存时间知晓率接近50%，人群对致肺癌危险的认知较好，了解吸烟和大气污染是肺癌的危险因素，但调查人群对肺癌筛查方法的认知不容乐观，X线筛查肺癌人群的知晓率仅有61.7%，痰细胞学，支气管镜以及低剂量CT的知晓率则更低，究其原因有三方面：未认识到筛查的好处，无症状不愿筛查，以及疾病负担，比例分别为63%、56.1%、66.5%。对早期肺癌的治疗，仅开胸治疗和化疗的知晓率达到50%以上，其他方法知晓率均较低。

### 2.3 肺癌防治知识知晓率影响因素分析

问卷中共涉及21个肺癌相关的防治问题，选择“是”说明调查对象知晓相对应问题的内容。调查对象对21个问题的知晓数目的分布情况见表 2。调查对象中，知晓6个以上的占80%，20个以上的仅占3%，其中知晓人数最多的在6题以上，其中6-12题的占42.6%，12-18题的占30.7%。调查人群中，未出现全部答对者，答对最多题目数量为19道，共10人答对。将21个问题中知晓数目小于等于9设为1，大于9设为0，分析不同影响因素之间的对知晓率的影响。

**1:** 肺癌防治知识总体知晓率情况 Overall awareness of lung cancer prevention and control

The content	Yes	No	Awareness rate (%)
The understanding of lung cancer			
Do you know lung cancer can be cured	1, 048	522	66.3
Do you know the early stage of lung cancer	1520	89	93.0
Do you know how many years people can live after early lung cancer being treated	664	943	40.6
Do you know your relatives with lung cancer take part in the census actively	884	714	54.1
Total	4, 116	2, 268	64.5
The risk factor for lung cancer			
Do you know smoking often can cause lung cancer	1, 233	373	75.5
Do you know secondhand-smoking can cause lung cancer	1, 225	382	75
Do you know air pollution can cause lung cancer	1, 371	238	83.9
What do you think of smoking ban can prevent lung cancer	1, 124	482	68.8
Total	4, 953	1, 475	77.1
The method of early lung cancer check			
Do you know X-ray can find early lung cancer	1, 008	601	61.7
Do you know sputum cytology can find early lung cancer	589	1, 020	36
Do you know bronchoscope can find early lung cancer	467	1, 142	28.6
Do you know LDCT can find early lung cancer	745	863	45.6
Total	2, 809	3, 626	43.7
The reason for refusing low dose CT screening			
Do not know the screening benefits	1, 029	577	63
Doing bronchoscopy is uncomfortable	606	1, 000	37.1
Disease psychological burden	918	688	56.1
No symptoms	1, 087	519	66.5
Don’t want to check	706	900	43.2
Be afraid cheated	430	1, 176	26.3
Total	4, 776	4, 860	49.6
Lung cancer treatment			
Do you think of opening thoracic surgery can treat early lung cancer	985	623	61.3
Do you think of chemotherapy can treat early lung cancer	944	664	57.8
Do you think of radiotherapy can treat early lung cancer	618	990	37.8
Total	2, 547	2, 277	52.8
LDCT: low dose CT.			

**2:** 肺癌肿瘤防治知识知晓数目情况 Number of awareness item about lung cancer prevention and control

Number of awareness	Number of people	Percent(%)
1-6	314	19.2
6-12	696	42.6
12-18	501	30.7
18-	48	2.9

为分析肺癌防治知识可能的影响因素，以肺癌防治知识知晓数目为因变量，对调查对象教育程度，BMI指数，吸烟情况，疾病史，家庭收入影响因素进行了分析。单因素结果显示年龄、教育、吸烟包年以及是否患有肺结核等对肺癌知晓情况有影响见表 3。

**3:** 肺癌防治知识知晓情况的单因素*logistic*回归分析 Univariate analysis of influencing factors of lung cancer awareness

Influencing Factors	OR	95%CI	*χ*^2^
Educational background			
Primary School or Below	1		5.319
Junior high school and above	0.758	0.599-0.959	
Smoking (package-year)			
-29	1		4.517
30-	1.268	1.018-1.580	
Age			
-55	1		5.384
56-	1.291	1.040-1.602	
Pulmonary tuberculosis			
Yes	1		5.607
No	2.329	1.134-4.784	

对单因素分析获得的有意义的变量纳入多因素*logistic*回归分析，最终进入多因素模型中的因素包括教育程度、吸烟、年龄、肺结核史见表 4。

**4:** 影响肺癌防治知识知晓情况的多因素logistic回归分析 Multivariate analysis of influencing factors about lung cancer awareness

Influencing factors	OR (adjust)	95%CI
Educational background		
Primary School or Below	1	
Junior high school and above	0.678	0.53-0.867
Smoking (package-year)		
-29	1	
30-	1.286	1.031-1.604
Age		
-55	1	
56-	1.43	1.141-1.791
Pulmonary tuberculosis		
Yes	1	
No	2.473	1.199-5.101

### 2.4 调查对象肺癌筛查意愿相关分析

本次调查人群对肺癌筛查的意识较好，能接受每年1次筛查的比例达到了80.3%；人群筛查所能承受的费用从免费到几千元不等，有效调查人数1, 624人，缺失9人，65.4%的调查对象能承受的筛查费用在500元以内，仅有25.2%的人群能承受500至1, 000的筛查检费用，能承担1, 000以上的调查对象则更少，仅有8.8%（表 5）。为分析肺癌筛查意愿的影响因素，我们将接受2年以上筛查的人群设为1，接受1年1次和2年1次体检的人群设为0，进行多因素*logistic*分析，结果表明性别、家庭收入、肺癌知晓情况以及教育程度是调查对象筛查意愿的影响因素（表 6）。

**5:** 调查对象肺癌筛查频率和体检费用承受能力情况 Acceptable frequency and payment willingness of health examination in respondents

Question	Frequency	Number	Percent(%)
The frequency of lung cancer screening you can accept	Once a year	1, 312	80.3%
	Once ever two years	230	14.1%
	Once every three years	36	2.2%
	Once every five year	55	3.4%
The cost you can bear	0-500	1, 068	65.4%
	500-1, 000	412	25.2%
	1, 000-1, 500	13	0.8%
	1, 500-10, 000	131	8%

**6:** 影响调查对象筛查意愿的多因素logistic回归分析 Multivariate analysis of influencing factors of the willingness of health examination

Influencing factors	*P*	OR^*^	95%CI
Age (year)			
-55		1	
56-	0.022	2.009	1.105-3.651
Gender			
Male		1	
Female	0.002	0.458	0.28-0.749
Income (0.1 M)	< 0.001		
-1		1	
1-2	0.155	0.335	0.074-1.511
2-3	0.595	1.176	0.647-2.139
3-	< 0.001	0.287	0.158-0.52
Number of awareness			
>9		1	
≤9	0.002	2.06	1.304-3.253
^*^Adjust the marital status, smoking, drinking, family history of cancer, lung disease and other chronic diseases.			

## 3 讨论

近30年来，肺癌已成为了全世界范围内的关注的癌症类型^[8]^。由于我国烟草流行率全球最高以及老龄化的影响，我国肺癌发生率和死亡率上升迅速，在本世纪开展的第三次死因回顾调查则显示肺癌已居癌症死亡原因首位^[9]^。作为二级预防措施，有效的筛查和早诊早治是改善肺癌生存、降低肺癌死亡率的关键。

LDCT检出肺癌能力明显高于X线胸片，其检出的肺癌生存明显改善，此外随机对照研究已证明LDCT检查真正降低了肺癌的死亡率^[10-12]^，一些医学组织已更新了肺癌筛查指南，建议在高危人群中开展LDCT肺癌筛查^[13-15]^。但LDCT的应用仍有一些不足之处，例如假阳性率高、过度诊断、辐射风险以及筛查成本较高等^[16-20]^。我国已于2009年起在天津大港和云南宣威开展利用LDCT进行肺癌高危人群筛查的试点工作。本次调查是2010年1月-2011年6月期间卫生部肺癌早诊早治大港项目点的基线调查，该调查有助于了解肺癌筛查人员对肺癌的认识程度，并对影响知晓率的因素进行分析，从而有助于更有针对性采取健康教育措施，降低肺癌发生率，提高肺癌人群的生存质量。

### 3.1 肺癌防治知识的总体知晓情况

本次调查得出：调查者了解肺癌的早期阶段的知晓率为93%，认为肺癌能治好的知晓率为66.3%。在我国第三次全死因调查中，肺癌死亡率最高，由于近年来，肺癌一直高发，人群中肺癌的知晓率也有所增加。被调查者认为大气污染能致肺癌的知晓率达到83.9%，吸烟能导致肺癌的知晓率仅为75%左右，认为戒烟能预防肺癌的知晓率则更低仅有68.8%，吸烟、空气污染、职业暴露、既往肺部疾病史、低水果摄入及遗传易感性等均为肺癌的危险因素^[21-28]^，但是吸烟是目前公认的肺癌最主要的危险因素，研究显示吸烟能解释中国75%的男性肺癌成因以及18%的女性肺癌成因^[29]^。另外该调查人群教育水平以初中为主，61.7%的人知道用X线检查早期肺癌，对于痰细胞学，支气管镜，CT的可用于筛查知晓率均不足50%。该调查得出人们不愿参加筛查的主要原因有三个：不了解筛查的好处、未出现症状、对疾病的恐慌，调查对象对肺癌治疗的认识主要集中在开胸手术。综合来看大港油田的工人对肺癌危险因素认识不全，筛查方法的知识欠缺，体检筛查意识较差。

### 3.2 影响肺癌防治知识知晓率的相关因素

对调查问题的知晓情况进行量化后，将可能影响肺癌知晓率的因素进行分类比较，得出不同年龄，不同教育程度，吸烟情况之间存在统计学差异（*P*>0.05），结果显示小于55岁的年龄组调查人群知晓率较高，初中及以上调查问卷知晓率高于小学及以下组。本调查将可能影响肺癌防治知晓率的相关因素进行*Logistic*回归分析，教育程度、吸烟、年龄、肺结核史是影响肺癌防治知识的知晓的因素，教育程度越高肺癌防治知识的知晓率越高；而吸烟是知晓水平的危险因素，吸烟包年越多肺癌防治知识知晓率越低。李星华等^[30, 31]^在中山市的鼻咽癌高发现场的研究表明，年龄因素、受教育程度影响人群对癌症防治知识的知晓情况，向群英等在湖北五峰宫颈癌高发区女性群体中的研究显示，受教育程度、肿瘤家族史和年龄与当地女性肿瘤防治知识知晓率相关，低学历、低收入、无肿瘤家族史、高龄人群的知晓率较低。山西阳城县食管癌筛查人群癌症防治知晓率的主要影响因素是性别、家族史和家庭人均收入，而广东梅州及湖南慈利宫颈癌筛查人群的研究则表明年龄越低、教育程度越高的个体癌症防治知晓率越高，本研究与其他类似研究差异的原因除肿瘤种类，如年龄段、性别、区域不同外，也与分析的具体过程和方法有关，但这些研究结果均显示教育程度高人群恶性肿瘤防治知识知晓率就高^[32-34]^。因此，天津大港地区应根据实际情况加大对低教育程度人群的肺癌防治知识宣传教育力度，鼓励高危人群定期体检，监测肺癌的高发。

### 3.3 调查对象肺癌筛查意愿

有效的肺癌早期检测和筛查能够降低肺癌死亡率并提高生存质量^[35]^。在本研究中调查对象无症状而不参加筛查的人达到了66.5%，亲属中积极参加筛查的比例也仅有54.1%，60%的调查人群不知道肺癌的预后情况。大港地区作为肺癌高发的地区，防治意识淡薄，体检意愿较低，应加强重视。

调查中显示绝大多数调查者（80.3%）会选择1年1次的筛查方式，2年1次仅占14.1%，3年及以上则更少，这说明该调查人群健康筛查的意识较强。65.4%调查者能承受的筛查费用在为0元-500元之间，25.2%能承受500元-1, 000元。由此可见大部分人群更愿意承受免费或者低费用的筛查，因此以后开展预防筛查项目应充分考虑体检地区的经济收入水平及承受能力，同时应加大资金的投入，更有效的开展健康教育。由多因素*logistic*回归分析得出，影响肺癌筛查意愿的因素为年龄，性别，家庭年平均收入以及肺癌防治知识知晓情况。分析得出，女性调查者以及更筛查意识更强，调整后的OR值为0.458。肺癌防治知识知晓率越低，筛查意识越差，知道9及以下条防治知识的危险度是知道9条以上的2.06倍，而家庭年平均收入也对筛查意识产生了很大影响，年收入3万元以上的家庭，更愿意参加筛查，由此看来，肺癌防治健康教育意义重大，癌症的防治不仅受我们生活环境的影响同时也受经济的制约。

在我国，癌症预防与控制规划纲要（2004年-2010年）中明确将“开展健康教育，提高公众对癌症主要危险因素的知晓率”作为癌症预防与控制的主要工作内容^[36]^。天津地区作为肺癌的高发地区，应积极开展肺癌以及恶性肿瘤宣传教育，提高广大人群的防癌意识，特别应加强特殊工种，年龄较大人群的防癌教育，同时应努力提高天津地区人群的就诊意识，在开展肺癌预防项目时，应充分考虑当地的经济水平，利用有限的资源提高人群的肺癌早诊早治意识，降低肺癌的发病率与死亡率。
